# Supplementary figures and images for: Development, physicochemical characterization and in-vitro biocompatibility study of dromedary camel dentine derived hydroxyapatite for bone repair
Source: PeerJ. 2023 Aug 3;11:e15711. doi: 10.7717/peerj.15711 (PMC10404400; doi:10.7717/peerj.15711)

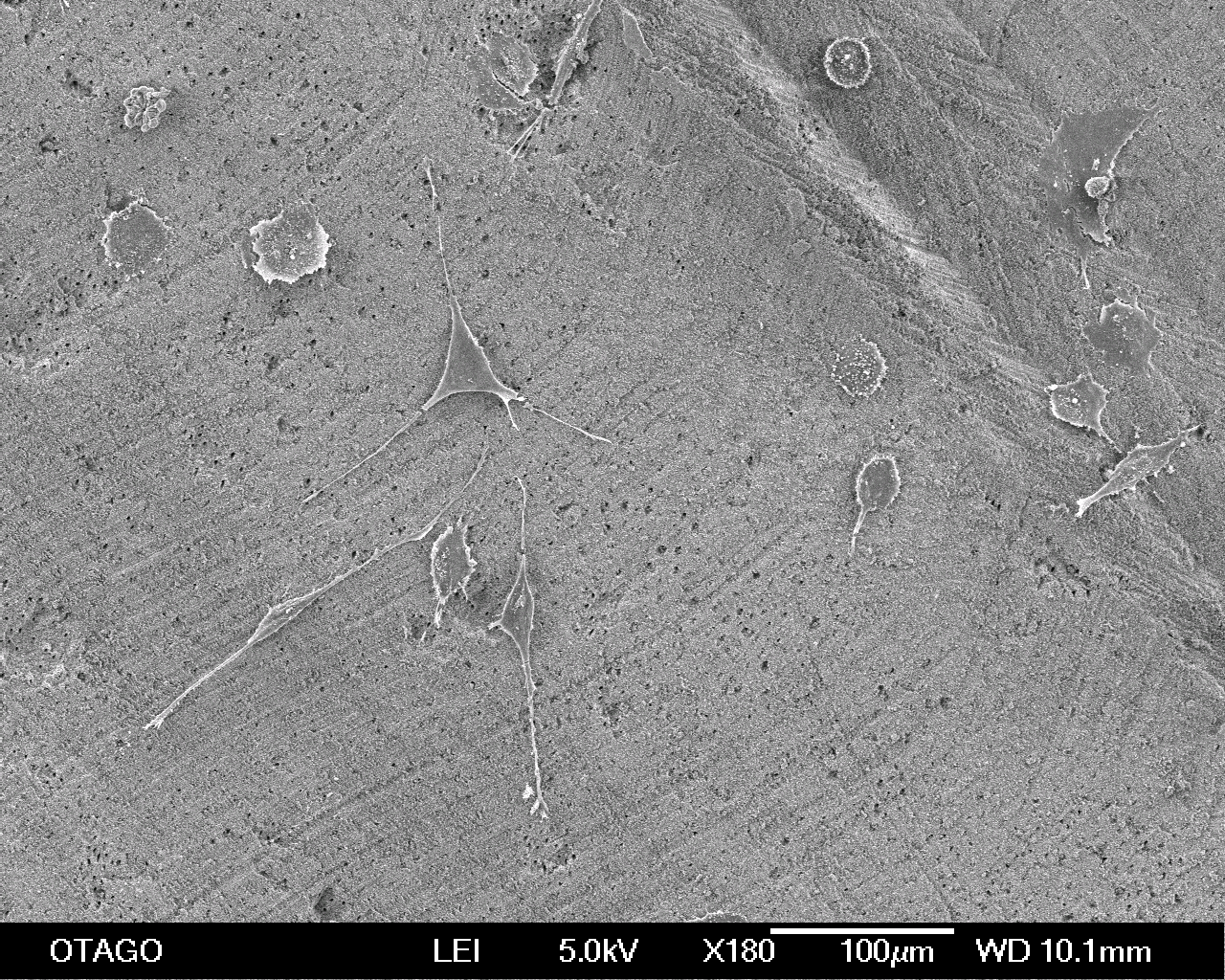

Supplement: Supplemental Information 4 [file peerj-11-15711-s004.tif]

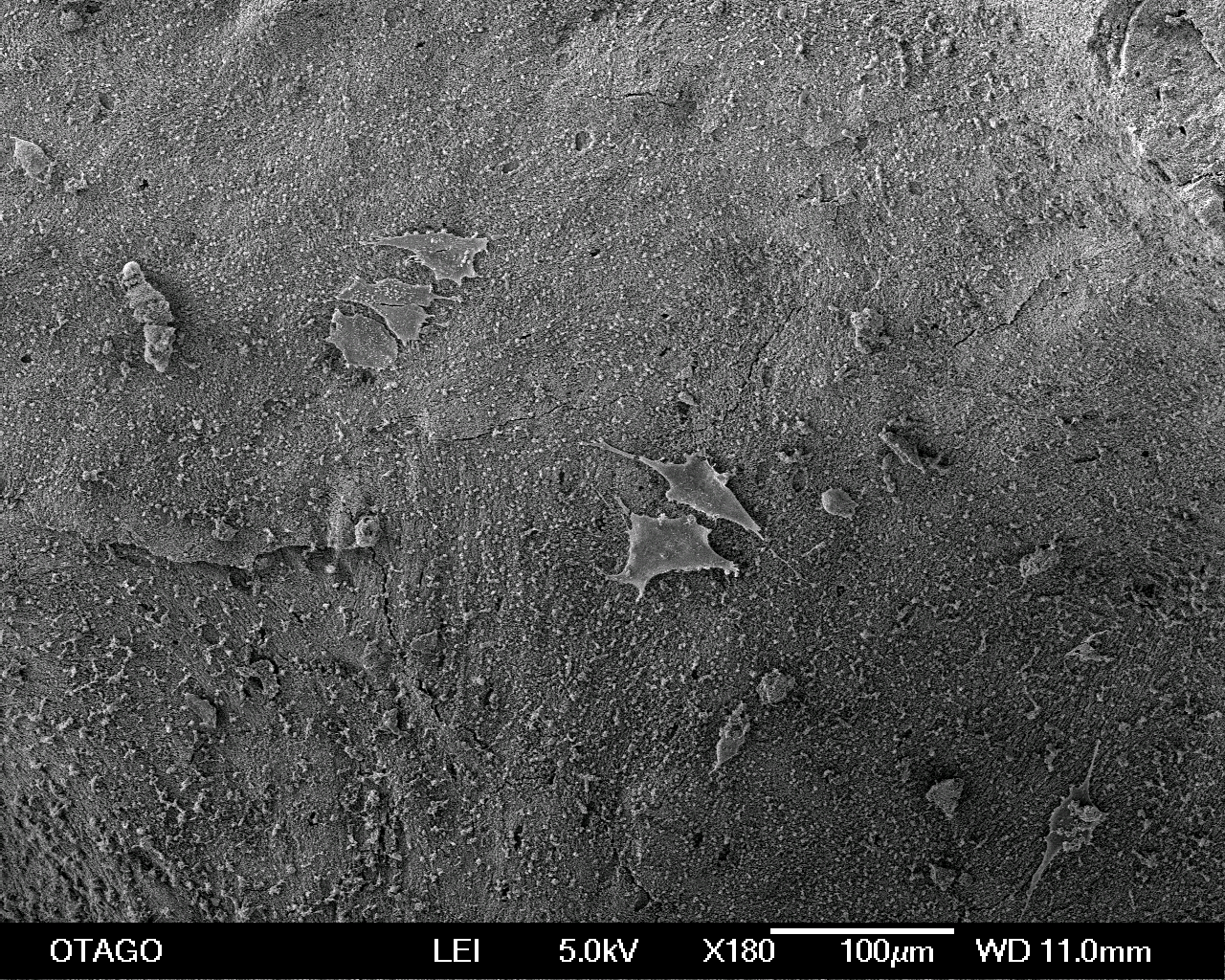

Supplement: Supplemental Information 5 [file peerj-11-15711-s005.tif]
